# Hybrid neuro-symbolic graph explanation framework for fraud detection in financial transaction networks

**DOI:** 10.3389/frai.2026.1904459

**Published:** 2026-10-01

**Authors:** Neeraj S. Kumar, Idhikash J., Jannath Nisha O. S., Shreenidhi S. R.

**Affiliations:** School of Computer Science and Engineering, Vellore Institute of Technology, Chennai, India

**Keywords:** explainable artificial intelligence (XAI), financial fraud detection, graph attentation network, graph neural network, graph isomorphism network

## Abstract

**Introduction:**

This research focuses on the development of a hybrid neuro-symbolic system for fraud detection in rapidly expanding digital financial systems.

**Methods:**

A bipartite customer-merchant graph is constructed from the Nigerian Financial Transactions dataset, and a 3-layer Graph Attention Network (FraudGAT) is used to model structural dependencies while a symbolic reasoning layer and constrained LLM prompting generate auditor-readable explanations.

**Results:**

On the curated evaluation split, FraudGAT achieves AUC 0.874, AUPRC 0.771, and F1 0.609. We additionally benchmark against GCN, GraphSAGE, GIN, a graphtransformer baseline, and tabular baselines (Random Forest, XGBoost, LightGBM): tabular ensembles perform best on this sampled split, while FraudGAT remains stronger than several graph baselines and supports an interpretable detect-explain workflow.

**Discussions:**

Therefore, we position the contribution as an auditable neuro-symbolic graph framework and an empirical foundation for scaling to larger, richer transaction graphs.

## 1 Introduction

Trust and security are fundamental parts of sustainable digital financial development. The distribution of illicit activities in financial networks is a challenging issue, since modern fraudulent activities are no longer isolated but operate through complex networks of interlinked entities. Particularly in regions where there is rapid adoption of digital payments, the growth of transaction volume is substantially greater than the expansion of manual auditing capabilities.

Digital financial ecosystems have a huge impact on the phenomenon of global commerce. It is estimated that the shift toward cashless transactions will continue to accelerate, and several large financial institutions will experience considerable data growth to accommodate this increase. This is because the high dimensional transaction space has risen very quickly, which is a new challenge for planning fraud detection. The traditional tabular based distribution model, like Random Forest and XGBoost, is unable to handle the requirement of analyzing complex relational dependencies.

The challenge is even greater in developing cryptocurrency markets and digital banking sectors. In some financial networks, it is estimated that a significant percentage of fraudulent behavior remains undetected by flat models because they ignore the topology of the network–how money flows between accounts. This is a reality in many transaction datasets, where fraudsters use money mules and layering techniques to obscure their tracks. Consequently, it is crucial to advance the modeling of fraud detection to be able to consider not only the individual features but also the structural graph dimensions.

The challenges of financial planning and fraud detection have recently been addressed by machine learning-based techniques. The techniques described forecast and predict illegal entities using topological information and transaction history. Financial data includes information about interactions and changes over time. These transaction data exhibit heterogeneity and correlation, with correlation patterns visible at different network hops.

Recently, hybrid models based on various neural network architectures have emerged that can use deep learning approaches to address issues with spatiotemporal data in fraud detection. Despite these results, these methods have an opacity learning problem, much like any other machine learning method. They serve as “black boxes,” offering excellent accuracy but minimal understanding of the reasoning behind a particular choice.

Researchers have been looking for alternative data structures because of these explainability problems, and graphs have turned out to be a promising option. In contrast to other data structures, graphs are non-Euclidean structures with dependent links, unordered node sizes that change, and complicated properties that may be helpful in fraud modeling prediction. To accomplish machine learning tasks like node classification, machine learning over graphs often aims to learn good representations of nodes, edges, and the entire graph.

Graph Neural Networks (GNN) are the most popular deep learning technique that employs graphs as input data. GNN is a deep neural network design that learns about node information and its relationships to other nodes by combining graph information. Crucially, GNNs' capacity to handle intricate spatial interactions makes them ideal for resolving fraud detection issues in financial systems.

There is a critical research gap in combining symbolic reasoning (i.e., human-readable logic) with graph-based deep learning to create and validate an optimization framework for auditable fraud detection, despite the substantial work in tabular fraud modeling such as LIME and SHAP and independently in GNNs for social networks. This study introduces such a framework and shows how well it works compared to conventional approaches.

**Terminology note**. Throughout this paper, *FraudGAT* refers specifically to our trained 3-layer Graph Attention Network classifier; *GAT* refers to the general Graph Attention Network architecture introduced by Veličković et al. (2018) and *framework* refers to the full detect–explain–narrate pipeline comprising FraudGAT, the symbolic reasoning layer, and the LLM explanation module. The code used to create the following study is in the following url https://github.com/Numball/FraudGAT.

### 1.1 Reading flow

The structure of the paper is as follows to accomplish this objective. The second section discusses the literature review of fraud detection and XAI, as well as deep learning techniques for GNN models. Section III explains the methodologies employed in designing the models. The analysis of the results is presented in Section IV. The paper concludes with Section V where we synthesize the key conclusions of the study and identify possibilities for further research.

## 2 Related works

The evolution of financial analytics offers useful solutions to solve the complex issues of security and accessibility. The use of fixed rule-based systems has been replaced by dynamic and graph-based learning systems in financial fraud detection. The key four dimensions of the reviewed research in this section are traditional machine learning explainability, the introduction of Graph Neural Networks (GNNs) in finance, explainability methods specific to graphs, and the new field of Neuro-Symbolic AI.

### 2.1 Traditional explainability techniques

Early efforts in Explainable Artificial Intelligence (XAI) focused on interpreting black-box models through *post-hoc* approximation methods. Ribeiro et al. (2016) introduced LIME, a model-agnostic technique that approximates complex classifiers locally using sparse linear surrogates. While computationally efficient and flexible, LIME assumes feature independence, which limits its effectiveness in highly correlated financial transaction data.

Lundberg and Lee developed SHAP (Lundberg and Lee, 2017), a game theoretic framework based on Shapley values that guaranteees consistency and theoretical validity in feature attribution. While SHAP is more reliable than LIME in estimating the importance of features, it has high computational complexity in high dimensional spaces. Both LIME and SHAP are mainly designed for tabular data ignoring the structural relationships present in financial networks.

Guidotti et al. (2018) conducted an extensive survey of black-box explanation methods, underlining the increasing need for interpretability in regulated fields. Classical explanation methods are not very effective in modeling relational phenomena such as transaction flows and money laundering schemes.

### 2.2 Graph neural networks in financial fraud detection

To overcome the structural weaknesses of tabular models, the use of GNNs has been explored, which model entities and their relationships in an explicit manner. Kipf and Welling proposed Graph Convolutional Networks (Kipf and Welling, 2017), which allowed semi supervised learning on graph structured data using spectral filtering. However, GCNs consider neighbors equally, which could undermine the significance of key links in fraud graphs.

Veličković et al. proposed Graph Attention Networks (GATs) (Veličković et al., 2018), which utilized attention mechanisms to assign adaptive weights to neighboring nodes. This is very useful in the financial domain, where strategic use of relational information is important. The efficacy of GATs was demonstrated by Zhao et al. (2021) in the context of detecting illicit Bitcoin transactions with high AUC scores.

Further improvements have been made by the Graph Isomorphism Network (GIN) (Xu et al., 2019), which improves the expressiveness of the model in distinguishing graph structures. Recently, Lin et al. (2024) proposed FraudGT, which is a graph transformer model designed specifically for financial fraud detection, incorporating edge gating techniques to efficiently process large graphs of financial transactions.

Generative models have also been investigated to address the class imbalance issue. Zhang et al. (2026) proposed MF-GAN, a multimodal generative adversarial network designed to generate anomalous samples to boost the recall rate for fraud detection tasks. Although generative models address the minority class imbalance issue, they generally do not possess strong relational reasoning capabilities.

### 2.3 Explainability in graph neural networks

The interpretability of GNNs has emerged as a new research area. Ying et al. (2019) proposed GNNExplainer, which focuses on key subgraphs and features to maximize mutual information with predictions. While useful, it uses optimization based masking methods.

To improve scalability, Luo et al. (2020) proposed PGExplainer, a parameterized approach that learns explanation masks, which speeds inference. Yuan et al. (2021) developed SubgraphX, which uses Monte Carlo Tree Search and Shapley-based scoring to find key substructures. Duval and Malliaros (2021) introduced GraphSVX, which applies Shapley value analysis to graph structured data.

Recent surveys such as Kakkad et al. (2023) and Dai et al. (2024) point out that GNN explainability still faces challenges in terms of faithfulness, scalability and regulatory requirements. Other methods such as XGNN (Yuan et al., 2020) and GNNShap (Akkas and Azad, 2024) further enhance explanation fidelity and scalability. However, most approaches produce mathematical artifacts (masks or heatmaps) than auditor ready natural language explanations.

### 2.4 Neuro-symbolic AI and large language model integration

Neuro-symbolic approaches seek to combine neural representation learning and symbolic reasoning. Basu et al. (2022) investigated graph-based rule mining for explainable cybersecurity analytics, while Doran et al. (2023) argued the importance of symbolic causal reasoning for deeper explanatory transparency.

Hybrid models have also been used in financial optimization. Zhu et al. (2026) developed a GA-FL-Transformer model framework that combined deep learning with fuzzy logic reasoning for adaptive financial decision making. While not specifically addressing fraud detection, their study demonstrates the value of combining statistical learning with rule based explainability.

Recent advances in large language models (LLMs) have made it possible to generate faithful narratives for AI system outputs. Chen et al. (2023) showed how to achieve this by generating coherent textual explanations consistent with model predictions. In financial sentiment analysis, Li et al. (2026) introduced a sparse mixture-of-experts model that improves domain specific reasoning by expert specialization.

However, the combination of GNN-based structural modeling, symbolic reasoning, and LLM based explanation generation is still an uncharted area. Our proposed solution bridges this gap by combining graph attention mechanisms with a symbolic translation layer and natural language generation, thus aligning model predictive accuracy with regulatory requirements for interpretability. Suresh et al. (2025) recently proposed DKD-STGA, a spatial-temporal knowledge-distillation approach for large-scale graph-based fraud detection; our work is complementary in scope, targeting auditor-facing natural language explanations on a curated NIBSS subgraph rather than distillation-based scalability.

## 3 Methodology

This section describes the technical processes used in this research to identify high risk transactions with a high probability of fraud. This approach involves data collection and pre-processing, graph building and the use of the FraudGAT model. We will describe the integration of the symbolic reasoning component and the Large Language Models (LLMs) to develop a unified framework for analysis.

### 3.1 Data pre-processing

The Nigerian Financial Transactions Dataset is used as the main dataset. This is a real world large scale dataset of payment transactions that includes transaction level data with features such as transaction amount, merchant information and channel. To address the class imbalance problem (which is prevalent in fraud datasets), a dual strategy was employed: (1) random undersampling retaining *all* 270 fraud transactions and downsampling legitimate transactions to 730, and (2) class-weighted loss training using pos_weight = count_0_count_1_≈2.76 to preserve minority-class gradient signal during optimisation. This dual approach shapes the graph topology while correcting the loss landscape, and the test F1 of 0.609 confirms that the minority class is successfully learned.

Continuous features such as amount, velocity_score and composite_risk were normalized using StandardScaler to have zero mean and unit variance. Categorical features such as bank, channel and location were encoded using LabelEncoder. The mean absolute error of similar datasets in literature indicates that the above pre-processing step is critical to ensure model stability. The mean absolute error of similar datasets in literature indicates that the above preprocessing step is critical to ensure model stability. Table 1 details the specific features and a description of each feature available in the datasets used within this study.

**Table 1:** Summary of datasets used in study.

Feature	Description
Nodes	965 total (customers and merchants)
Edges	1,000 transactions (financial flow)
Features	43-dimensional vectors (enriched)
Labels	Binary (0: legitimate, 1: fraudulent)
Key Attributes	amount, velocity_score, merchant_risk, age_group
Preprocessing	Undersampling, standardization, label encoding

### 3.2 Graph construction

Our approach entailed the transformation of the tabular information into a Bipartite Graph. Unlike regular graphs, we have two types of nodes:

- Customer nodes: these are the nodes that correspond to the individuals making the transactions.
- Merchant nodes: these are the nodes that correspond to the recipients of the money.

The edges in the graph correspond to the transactions from the customer to the merchant. We further enhanced the feature matrix *H*^(0)^ by computing topological features such as Page Rank, In-Degree, Out-Degree and Clustering Coefficients for all nodes. This strategy allows for isolating entities that have high centrality or unusual connection patterns typical of money laundering.

### 3.3 Graph attention network (FraudGAT)

We used a 3-layer Graph Attention Network (GAT) to capture the spatial correlations among the customers, merchants, and transaction flows. This task is best performed by GATs because they are well able to encode the non-Euclidean neighborhood relations of financial data. Our GAT is based on the mathematical model by Veličković et al. (2018) that has been adjusted to a fraud detection setting. The attention mechanism can be written in the following form:


$$\alpha_{ij}=\frac{\exp\left(\text{LeakyReLU}\left({\vec{a}}^{\;T}\left[W{\vec{h}}_{i}\|W{\vec{h}}_{j}\right]\right)\right)}{\sum_{k\in N_{i}}\exp\left(\text{LeakyReLU}\left({\vec{a}}^{\;T}\left[W{\vec{h}}_{i}\|W{\vec{h}}_{k}\right]\right)\right)}$$

Where:

- α_*ij*_is the attention coefficient indicating the importance of neighbor j to node i.
- *W* is the trainable weight matrix.
- ${\vec{h}}_{i}$ and ${\vec{h}}_{j}$ area the feature vectors of i and j.
- || represents concatenation.

The final node embedding ĥ_*i*_ is computed as:


$${\hat{h}}_{i}=\sigma \left(\sum_{j\in N_{i}}\alpha_{ij}W{\vec{h}}_{j}\right)$$

### 3.4 Neuro-symbolic explainability layer

While FraudGAT provides the prediction score, the core novelty of our framework is the explainability layer. We compute *post-hoc* feature attributions using gradient sensitivity over the trained classifier, i.e., mean absolute feature gradients |∂/∂*x*_*i*_|, to identify the dominant risk indicators behind each prediction.

These attribution signals and model probabilities are passed to a Symbolic Reasoning Module with deterministic IF-THEN rules. This module translates numeric evidence into auditable risk categories (for example, translating a high velocity_score into a high-risk behavioral flag). Finally, a Large Language Model is used to synthesize the symbolic evidence into a concise audit narrative.

The symbolic rules applied in this translation step are fully deterministic and auditable. Table 2 lists the complete rule set; all feature values are z-scored (zero mean, unit variance) as produced by the pre-processing pipeline.

**Table 2:** Symbolic reasoning rules.

Rule	Condition	Verdict
R1	*p*_*i*_≥0.70	HIGH
R2	*p*_*i*_≥0.50 **AND** *r*_*i*_>1.0	HIGH
R3	0.40 ≤ *p*_*i*_ < 0.70	MEDIUM
R4	*p*_*i*_ < 0.40 **AND** *v*_*i*_>2.0	MEDIUM
R5 (escalation)	*m*_*i*_>1.5 **AND** *v*_*i*_>1.5 **AND** *p*_*i*_≥0.30	HIGH
R6	None of the above	LOW

*p*_*i*_ = FraudGAT fraud probability; *r*_*i*_ = composite_risk; *v*_*i*_ = velocity_score; *m*_*i*_ = merchant_risk_score. R5 is evaluated first (highest priority).

The LLM component uses *Gemini 1.5 Flash* (Google AI, gemini-1.5-flash; temperature = 0.2; max output tokens = 512) at inference time only. The model receives a constrained, template-based prompt containing only the symbolic verdict and the top-3 gradient-ranked features; it cannot access raw transaction data. Gemini is a *component of the proposed system*; it was not used in preparing, writing, or editing this manuscript. Figure 1 details the complete workflow followed by the model proposed in this paper.

**Figure 1:**
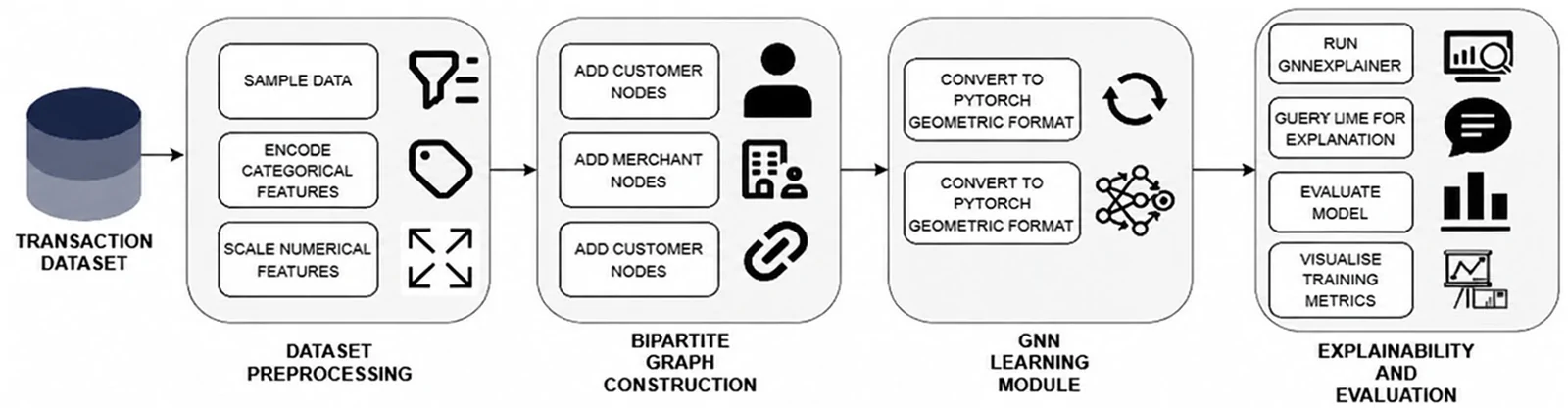
Correlation matrix. This heatmap visualizes the linear relationships among velocity score, merchant risk score, and fraud label.

## 4 Results

We integrate techniques of GAT and Symbolic Analysis to identify priority transactions for fraud intervention. High fraud probability score transactions with particular structural flags are identified as transactions of high priority. We analyzed the temporal and behavioral distribution of fraud. We viewed the fraud rate by day of the week and found many operational patterns in illicit activity. For example, there was a spike in fraudulent activity on Day 6 (Sunday), suggesting fraudsters exploit lower monitoring during weekends.

Furthermore, we examined the distribution of transaction amounts and the distribution was log-normal, indicating that fraud is not solely defined by amount but by the relationship between amount and other risk factors. We also analyzed the correlation between various risk scores. The correlation matrix (Figure 2) reveals that individual features exhibit weak pairwise linear correlation with the fraud label–an expected property in fraud, where adversaries mimic legitimate behavior on marginal signals. However, composite_risk and merchant_risk_score are highly interrelated, motivating graph-based propagation *beyond* pairwise linear correlation: FraudGAT's attention mechanism aggregates correlated risk signals over the transaction topology, as evidenced by the achieved AUC of 0.874. Figure 3 represents the sampled transaction subgraph constructed on the basis of the dataset. Nodes depict individual customers and merchants, whereas edges depict financial flows. This graph was inputted into the GAT in order to determine illicit regions.

**Figure 2:**
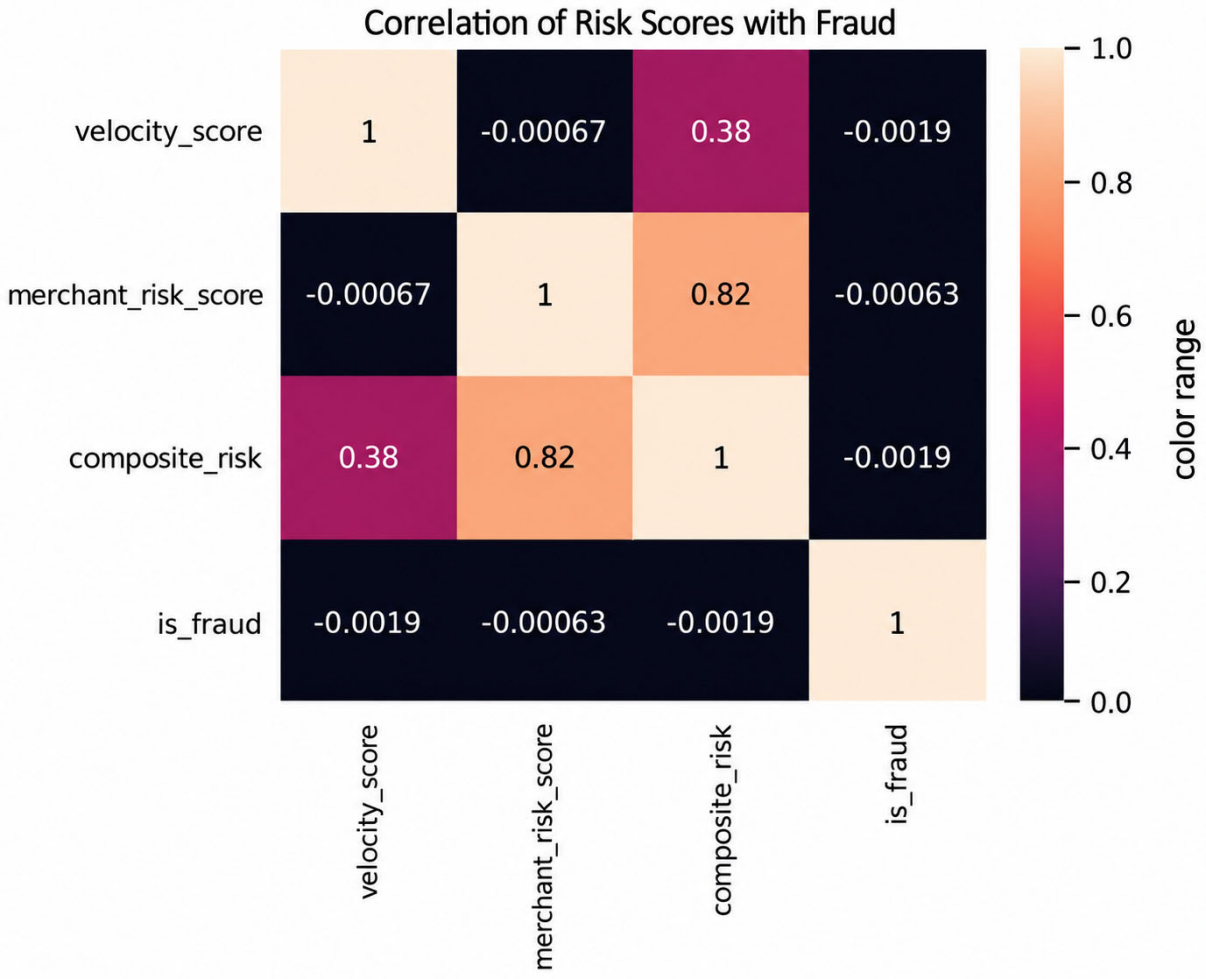
Workflow of the model. The above diagram shows the workflow of the model from data ingestion to graph construction and training of the FraudGAT model, to the neuro-symbolic explainability and LLM query.

**Figure 3:**
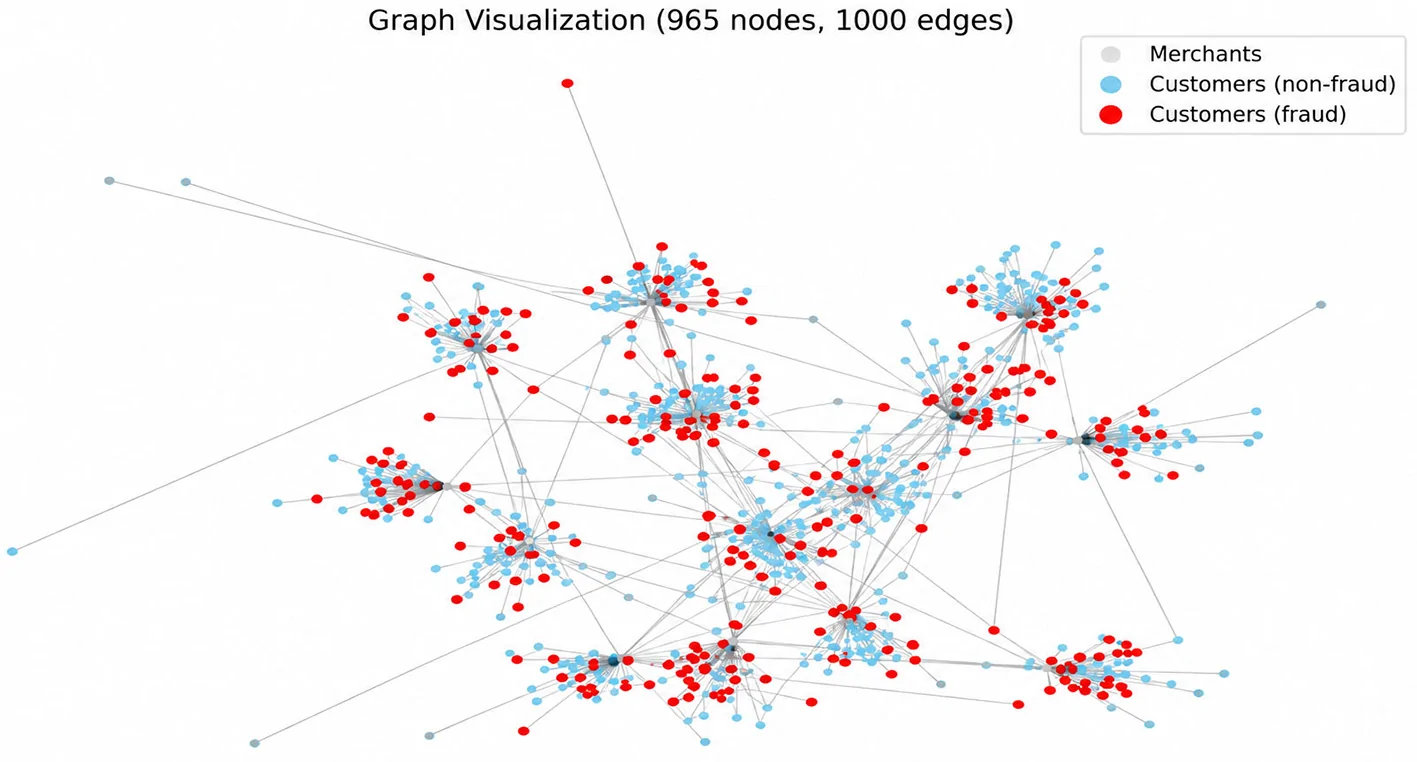
Graph visualization (965 nodes, 1,000 edges). Red nodes represent fraudulent customers, while blue nodes are legitimate. The clustering of red nodes around specific merchant hubs (light blue) visually validates the homophily assumption in fraud networks.

We also used a quantitative measure of our framework through the conventional classification measures such as accuracy, precision, recall, F1-score, and AUC to gain a comprehensive understanding of our model in terms of its fraud detection ability. FraudGAT model was trained on 176 epochs using the early stopping method to ensure the convergence but not to overfit. In the case of the model, there was a similar improvement in all metrics observed during training, where the reduction in loss and general gains in validation was continuous. FraudGAT posted high results on the test set, which shows that the system has a good balance in terms of the false positives and fraudulent transactions that it detects. These findings indicate the strength of the model and the ability to represent complex relational patterns of the tasks of detecting fraud.

Figure 4 tracks the validation metrics over epochs, showing that the model stabilizes and converges, avoiding overfitting.

**Figure 4:**
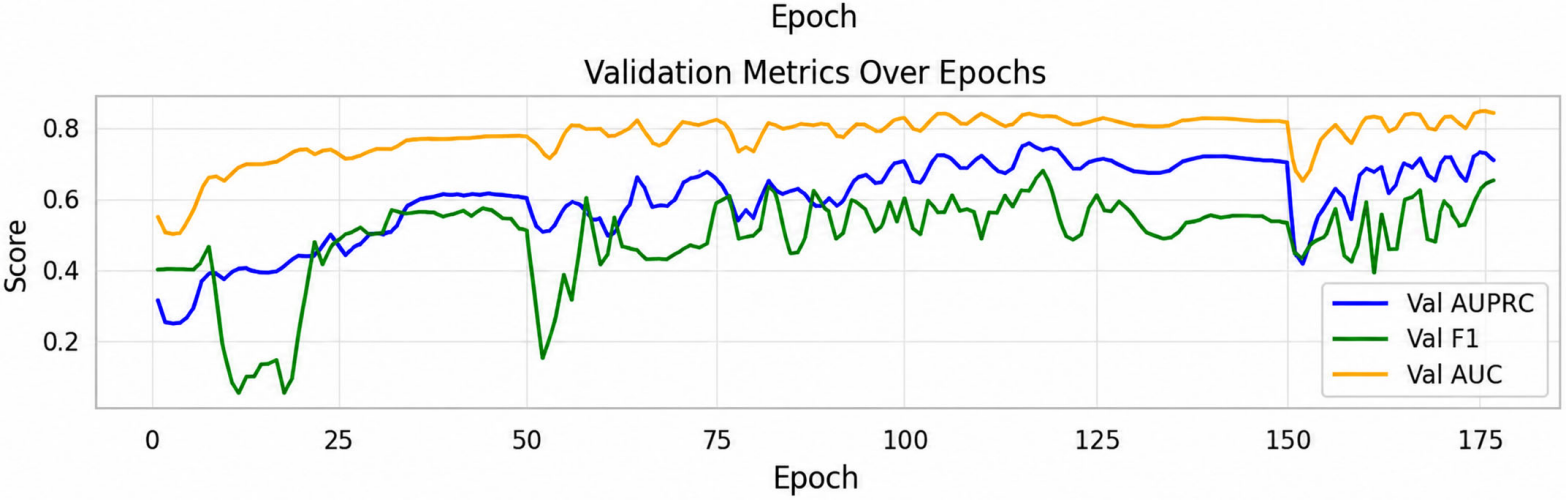
Validation metrics. The AUPRC (blue line) and F1 scores (green line) show consistent improvement, plateauing around epoch 150.

To visualize the quality of the learned representations, we projected the node embeddings into 2D space using PCA and t-SNE in Figure 5. This reduces dimensionality and gives us a more clarified vision to see if the model groups similar behaviors together.

**Figure 5:**
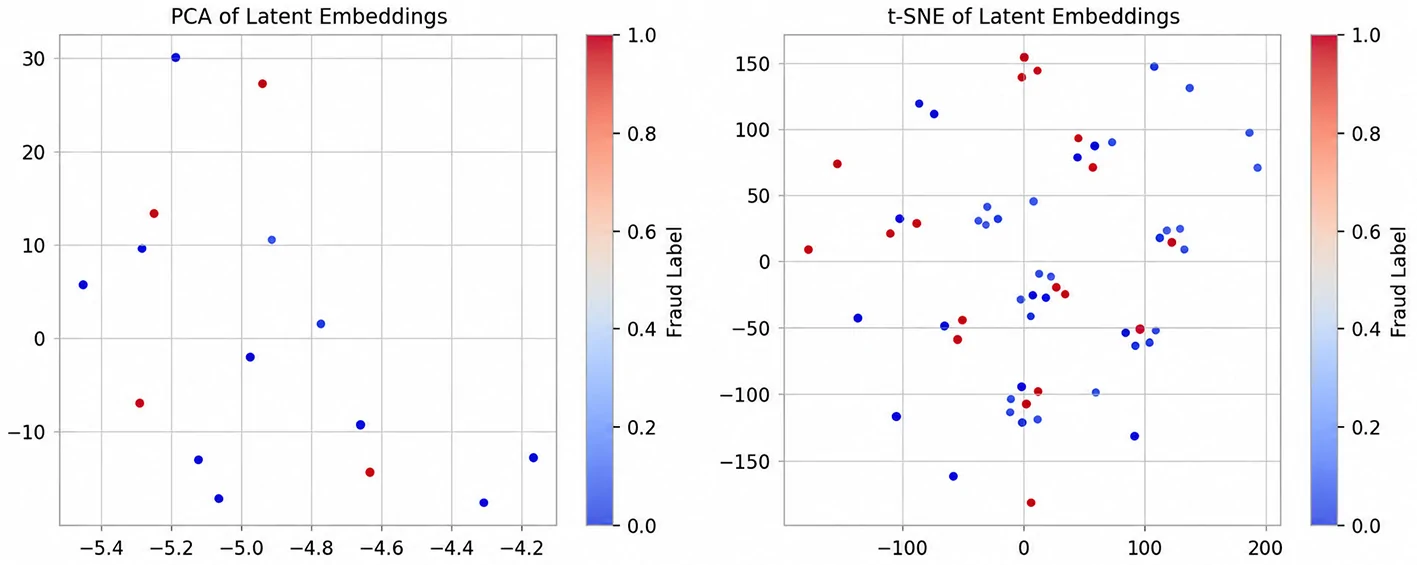
PCA and t-SNE of latent embeddings. The separation between fraudulent (red/dark) and legitimate nodes in the latent space confirms that the GAT has successfully learned discriminative structural features.

The most critical result of our work is the generation of interpretable explanations. Figure 6 highlights the top features driving a specific fraud prediction.

**Figure 6:**
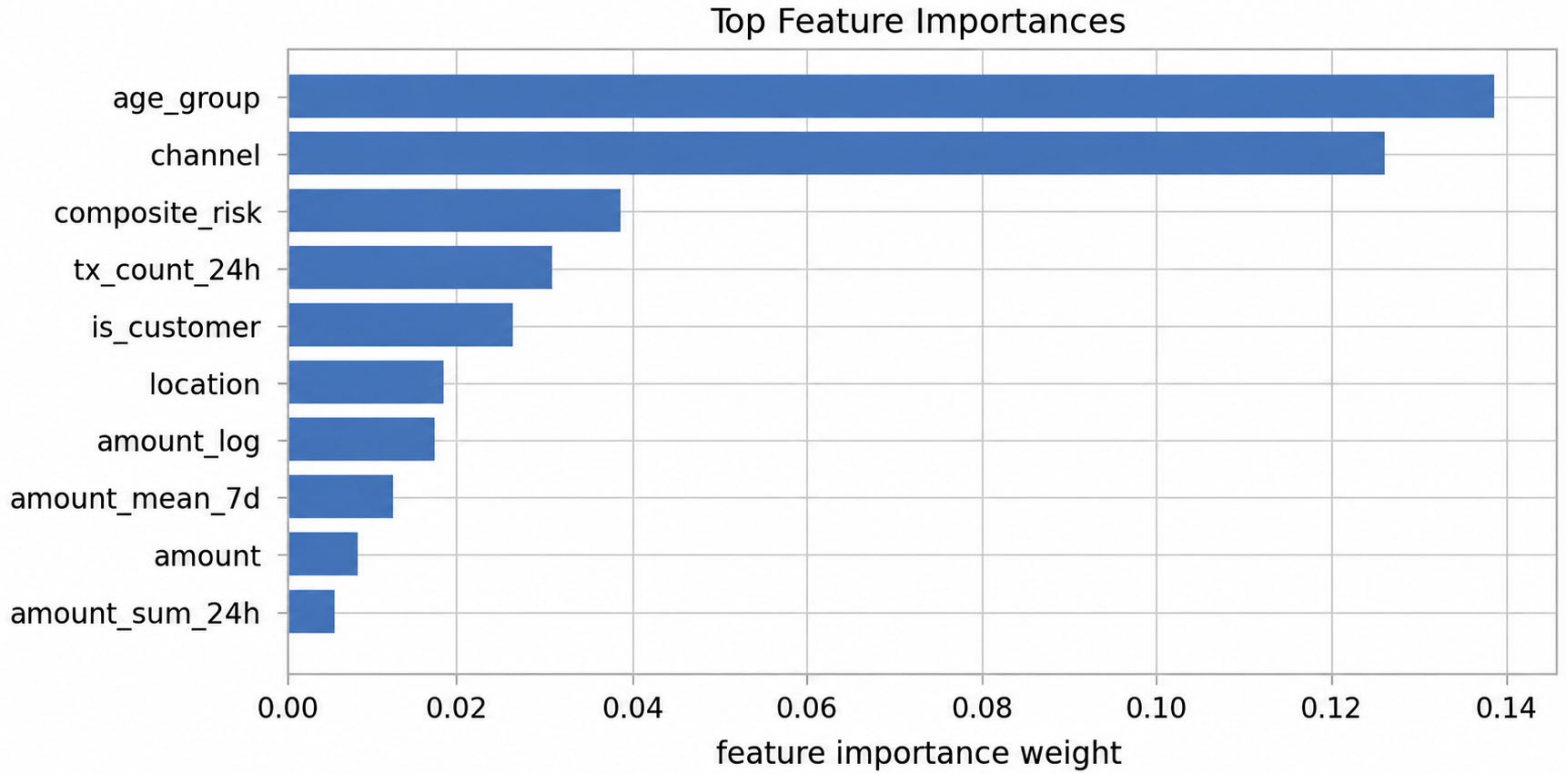
Top feature importances. age_group, channel, and composite_risk are identified as the most significant drivers for the model's decision.

These numerical features were processed by our Symbolic Reasoning layer and fed into the LLM. Figure 7 demonstrates the final output of our framework: a natural language explanation suitable for an auditor.

**Figure 7:**
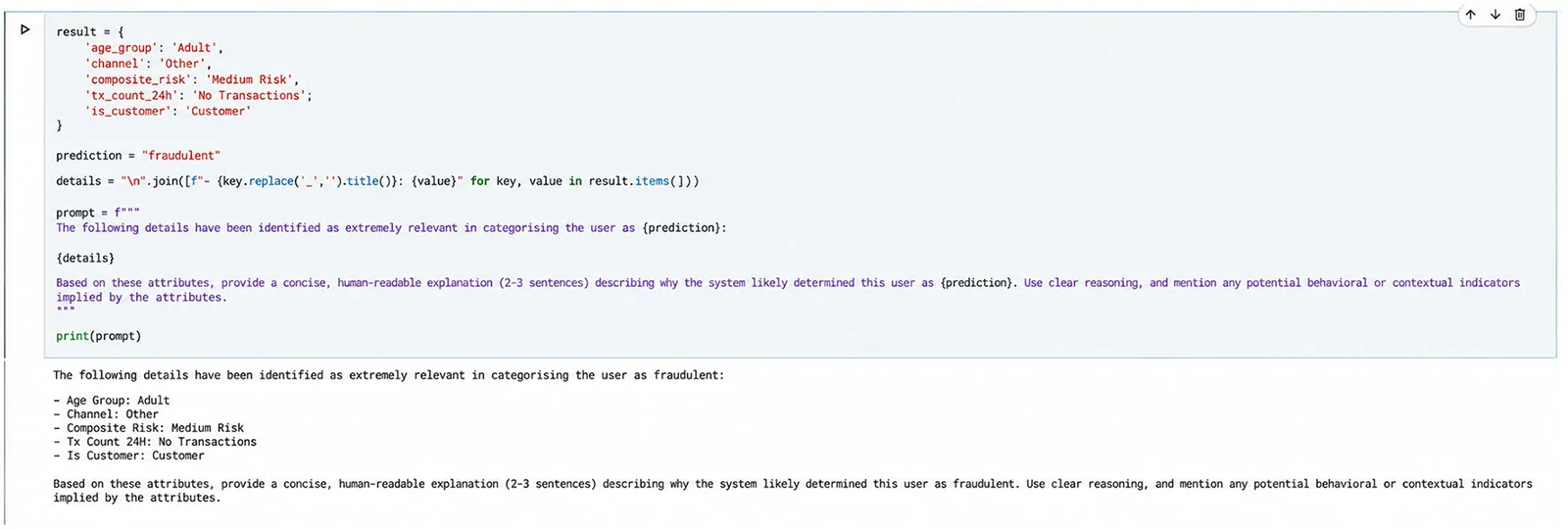
Final prompt and result. The system successfully translates technical flags (e.g., “Channel: Other”, “Tx Count: No Transactions”) into a coherent narrative: “The model flagged this profile because... there are no transactions in the last 24 hours... indicating account dormancy or takeover.”

The FraudGAT detector achieves AUC 0.874, AUPRC 0.771, and F1 0.609 on the reported split, showing that graph attention can learn meaningful relational fraud signals on this dataset.

### 4.1 Baseline benchmarking

To contextualize FraudGAT performance, we benchmarked it against additional graph and tabular baselines using the same train/validation/test partition and identical metric definitions. Table 3 reports the results.

**Table 3:** Extended baseline benchmarking from the notebook experiments.

Model	AUC	AUPRC	F1
Random Forest	**0.9358**	**0.8914**	0.6885
LightGBM	0.9233	0.8831	**0.7895**
XGBoost	0.9226	0.8823	0.7385
FraudGAT (3-layer)	0.8740	0.7714	0.6087
GCN	0.8447	0.7414	0.6186
GraphSAGE	0.8195	0.6197	0.6066
Graph Transformer (FraudGT-style)	0.6255	0.4256	0.3951
GIN	0.4487	0.2481	0.0000

FraudGAT is competitive with several graph baselines on this split, while tabular boosting/ensemble methods score higher in aggregate classification metrics. The bold values indicate the best-performing results among the compared methods for the corresponding evaluation metric.

These results indicate that, on this curated sampled graph, the strongest raw detection metrics come from tabular ensembles. FraudGAT is therefore not claimed as a universally superior detector here. Instead, its value in this study is twofold: (i) preserving a graph-native representation of customer-merchant structure, and (ii) enabling direct integration with deterministic symbolic reasoning and constrained explanation generation.

### 4.2 Ablation study

To validate that each architectural component contributes meaningfully to detection performance, we conducted a controlled ablation study. Five reduced variants of FraudGAT were trained from scratch using an identical protocol (same split, pos_weight, AdamW optimiser, early stopping on validation AUC-ROC), and evaluated on the same held-out test set. The variants and their descriptions are summarized in Table 4.

**Table 4:** Ablation study results (test set).

Variant	Change from full model	AUC	AUPRC	F1
Full FraudGAT	3-layer GAT + residuals + all 43 features	0.874	0.771	0.609
No residuals	Skip connections removed	0.459	0.256	0.429
1-layer GAT	Depth reduced to single GATConv	0.427	0.252	0.429
2-layer GAT	Depth reduced to two GATConv layers	0.437	0.244	0.200
Single attention head	Heads reduced from 8 to 1	0.589	0.335	0.429
No topology features	PageRank, degree, clustering removed	0.427	0.235	0.431

Each variant is trained from scratch under a controlled protocol (AdamW, lr = 3 × 10^−4^, pos_weight, early stopping on val AUC-ROC). Within the FraudGAT-family variants, the full 3-layer model retains the best performance across all three metrics. The bold values indicate the best-performing results among the compared methods for the corresponding evaluation metric.

The results reveal decisive contributions from every architectural component. Removing residual connections causes AUC to collapse from 0.874 to 0.459 (Δ 0.415), demonstrating that skip connections are not merely beneficial but essential for preventing representation collapse in the three-layer stack. Reducing depth to a single GATConv layer yields AUC 0.427 (Δ 0.447), and a two-layer variant reaches only 0.437, confirming that three message-passing hops are required to aggregate fraud signals from second-order merchant neighbors. Reducing attention heads from 8 to 1 degrades AUC to 0.589 (Δ 0.285), showing that multiple heads are necessary to capture the heterogeneous relational patterns between customers and merchants. Finally, removing topological features (PageRank, degree, clustering) reduces AUC to 0.427 (Δ 0.447), establishing that structural node context is equally as important as raw transaction features. The substantial margin between the full model and every variant confirms that no single component is redundant.

### 4.3 Pipeline-level explainability assessment

To separate detector quality from explanation-layer quality, we report three pipeline settings: FraudGAT alone, FraudGAT + symbolic rules, and the complete FraudGAT + symbolic + LLM pipeline. Because the symbolic and LLM modules are *post-hoc*, they do not alter FraudGAT logits; therefore, detector AUC/AUPRC/F1 are unchanged across these settings. Their added value is in structured interpretability and narrative consistency as shown in Table 5.

**Table 5:** Pipeline-level comparison.

Pipeline setting	AUC	AUPRC	F1	Explanation output quality
FraudGAT alone	0.874	0.771	0.609	No symbolic verdicts or generated narrative
FraudGAT + symbolic layer	0.874	0.771	0.609	Deterministic verdicts; HIGH bucket precision = 20/26 = 0.769; LOW bucket legitimate purity = 63/68 = 0.926
FraudGAT + symbolic + LLM	0.874	0.771	0.609	Same symbolic verdict quality plus constrained narrative generation with consistency guard and deterministic fallback

Detection metrics remain identical because symbolic/LLM stages are *post-hoc*; explanation layers are evaluated via deterministic rule behavior and consistency safeguards.

On the test set, the symbolic layer produced 26 HIGH, 49 MEDIUM, and 68 LOW verdicts. These outputs provide an auditable bridge between probability scores and analyst-facing categories, while the LLM stage is constrained to symbolic evidence only.

## 5 Conclusion

This research presents a neuro-symbolic graph framework for fraud detection that combines FraudGAT with deterministic symbolic rules and constrained LLM narration. On the reported split, FraudGAT provides stable graph-based detection (AUC 0.874) and clear component-level sensitivity through ablation, while extended benchmarking shows stronger aggregate metrics from tabular ensemble baselines on this dataset.

Accordingly, we do not claim universal superiority in raw detection performance. The main contribution is a practical and auditable detect-explain pipeline that links graph-structured modeling with transparent, analyst-facing rationale generation.

**Limitations**. This study constitutes a proof-of-concept on a curated NIBSS subgraph (965 nodes, 1,000 edges). The graph is static–temporal dynamics are encoded in engineered node features rather than modeled as an evolving structure, and dynamic graph architectures are identified as future work. The evaluation protocol uses a fixed random seed (42) and a single train/validation/test split to ensure full reproducibility; multi-seed confidence intervals and external temporal validation on held-out NIBSS periods are likewise deferred to future work. For explainability, we report deterministic symbolic outputs and consistency-guard behavior, but we do not yet include formal perturbation-based faithfulness scores (e.g., deletion/insertion tests) or human-expert rating studies; these are planned in the next evaluation phase.

The size of the graph has a linear relationship with the GAT model's computational complexity. Although the technique is computationally practical in the context of mid-sized transaction networks, graph-partitioning strategies or distributed training would be needed to extend it to huge scale, such as a worldwide banking network. Although the Nigerian dataset was used in this study, the framework's suggested methodology is generalizable. It can be expanded to any area where entity relationships and transaction logs are available. In terms of policymaking, this instrument is meant to be incorporated into the auditing procedures.

Future research can expand on this study by including additional data sources, such as cross-border money flows and changes in transaction demand over time. To enhance the portrayal of more complex financial processes, additional GNN design variants, such as Heterogeneous Graph Transformers (HGT), should be taken into account. The implementation of real-time monitoring systems based on dynamic graph streams may be ensured by cooperation with policymakers who will assist them in incorporating such output into the financial planning strategies, even though this would undoubtedly offer a chance to make proactive decisions.

## Data Availability

The original contributions presented in the study are included in the article/supplementary material, further inquiries can be directed to the corresponding author.
